# Shifting and zooming through space … or not: The role of attention in spatial compatibility tasks - A replication of Stoffer (1991)

**DOI:** 10.1007/s00426-026-02299-1

**Published:** 2026-05-04

**Authors:** Pamela Baess, Shabnamalsadat Ayatollahi, Christina Bermeitinger, Luisa Bogenschütz, Pia Fenske, Ryan P. M. Hackländer, Mohammad Hamzeloo, Gustavo Adolfo León Montoya

**Affiliations:** Department of Psychology, University of Hildesheim, Unversitaetsplatz 1, Hildesheim, D – 31141 Germany

**Keywords:** Simon effect, Spatial compatibility, Attentional shifting, Referential coding, Perception and action, Delta plots

## Abstract

The mechanism of spatial coding involved in the Simon effect remain debated, with two prominent theories – the attention-shifting account Stoffer, (*Psychological Research, 53*(2), 127–135, 1991) and the referential coding account Hommel, (*Psychological Research, 55*(3), 208–222, 1993b) – offering competing explanations why the irrelevant stimulus-location is coded. To date, neither account can fully explain all available empirical data. Most published studies rely on limited sample sizes, which constrains statistical power and leaves the field without conclusive evidence. This study directly replicated Stoffer’s (*Psychological Research, 53*(2), 127–135, 1991) investigation into attentional subprocesses that generate the Simon effect. We manipulated target spatial position across two reference frames: a global frame based on target’s screen side and a local frame defined by the relative position within each screen side, allowing us to test for both global and local Simon effects. Targets were presented inside precues, either a large box or two small boxes, which were presented simultaneously with or sequentially before the target. With an adequately-powered sample, our study revealed concurrent global and local Simon effects – a result different from Stoffer’s original findings. Only the local Simon effect varied with precue type and simultaneous or sequential target presentation. These results clarify the conditions under which attentional shifting *and* referential coding mechanisms contribute to different Simon effects, demonstrating that when spatial target positions are defined by both global and local reference frames, these mechanisms shape the emergence of distinct Simon effects in different ways.

**Supplementary Information:**

The online version contains supplementary material available at 10.1007/s00426-026-02299-1.

## Introduction

It has been a long-standing core interest of psychology to understand the role of attention in human performance (e.g. James, 1890). Despite over a century of interest, the matter is still far from being settled (e.g. Hommel et al., 2019). Attentional mechanisms are relied on to explain various findings. For example, selective attentional mechanisms are proposed in order to explain the failure in the detection of signal changes (Simons & Chabris, 1999) or the level of attentional processing (global or local) prioritized in visual perception (Navon, 1977). Selective attentional explanations have also been put forward to aid explaining spatial compatibility effects, including the original explanation of the Simon effect (Simon & Small, 1969).

In Simon and Small’s experiment (1969), the participants were unable to ignore the (task-irrelevant) spatial source of differently pitched-tones presented through one of two earphones. Participants moved a control handle to the left or right position depending on the ear in which they heard the tone. Performance was better when the stimulus and the required response location were compatible (e.g., a tone in the left ear and a required response with the left hand) than incompatible (e.g., a tone in the right ear and a required response with the left hand). The differences (labeled as Simon effect) in response times and/or accuracy between the compatible and incompatible trials indicate that spatial stimulus-response compatibility matters.

The Simon effect (for overview, Hommel, 2011; Proctor, 2011; Simon, 1990) and related spatial compatibility phenomena have been explained as arising from a conflict between competing spatial codes generated by two distinct processing pathways (De Jong et al., 1994; Kornblum et al., 1990). The conditional processing pathway activates the spatial representations based on the instructed task. For example in a visual Simon task, a square-shaped stimulus is assigned to a left response and a rectangle-shaped stimulus is mapped to a right response. At the same time, the spatial location of the stimulus (e.g., on the left or right screen’s side) activates an unconditional spatial code through a separate automatic processing pathway based on the stimulus’ location (De Jong et al., 1994). The interaction and cognitive conflict between these spatial codes leads to the Simon effect, reflecting how task-irrelevant spatial information influences response selection.

Although the Simon effect is well-documented, the precise mechanisms underlying the formation of spatial codes remain unclear (cf. Umilta, 1994). Two main perspectives have been proposed: the attentional-shifting account and the referential-coding account. The attentional-shifting account emphasizes the role of attentional processes in generating spatial codes relevant to the Simon effect (Nicoletti & Umilta, 1989, 1994; Stoffer, 1991; Stoffer & Yakin, 1994), underlining the relevance of a lateral attention shift towards the side of the imperative stimulus. Orienting attention toward the imperative stimulus produces the spatial code relevant for the Simon effect. Attention-shifting accounts of the Simon effect differ in regard to the processes following the lateral shift (Stoffer, 1991; Umilta & Nicoletti, 1992), however all consider the location before the lateral attention shift as the origin of the spatial reference frame. Interestingly, relative spatial stimulus coding is then seen as a by-product of the attentional orientation process.

In contrast, the referential-coding account focuses on the spatial code as formed relative to an intentionally defined object or reference frame (Hommel 1993b; van der Heijden et al. 1999), highlighting the availability of reference frames for the formation of spatial codes. Spatial location of the object serves as the reference for spatial coding, which may be the starting point for additional explorative attention shifts considering attentional focusing an epiphenomenon (cf. Stoffer & Umilta, 1997). According to this perspective, spatial coding – and thus the Simon effect – depends not on attentional mechanisms per se, but rather on the presence of a contextual reference against which stimulus position is coded. This idea suggests that attentional subprocesses are not at all involved but referential coding processes take place when a reference point is given.

Although some researchers have attempted to integrate these views (Proctor & Lu, 1994; Stoffer & Umilta, 1997; Umilta & Nicoletti, 1992), the question of how spatial codes are formed – based on lateral attention shifts or referential coding processes – remains open. Moreover, as both the attention-shift account and the referential coding account both rely on differences in coding processes as a result of attention orientation or reference objects, they are difficult to test independently. However, both accounts were postulated as alternative explanations for the absence of a Simon effect in the study by Umilta & Liotti (1987).

**Fig. 1:**
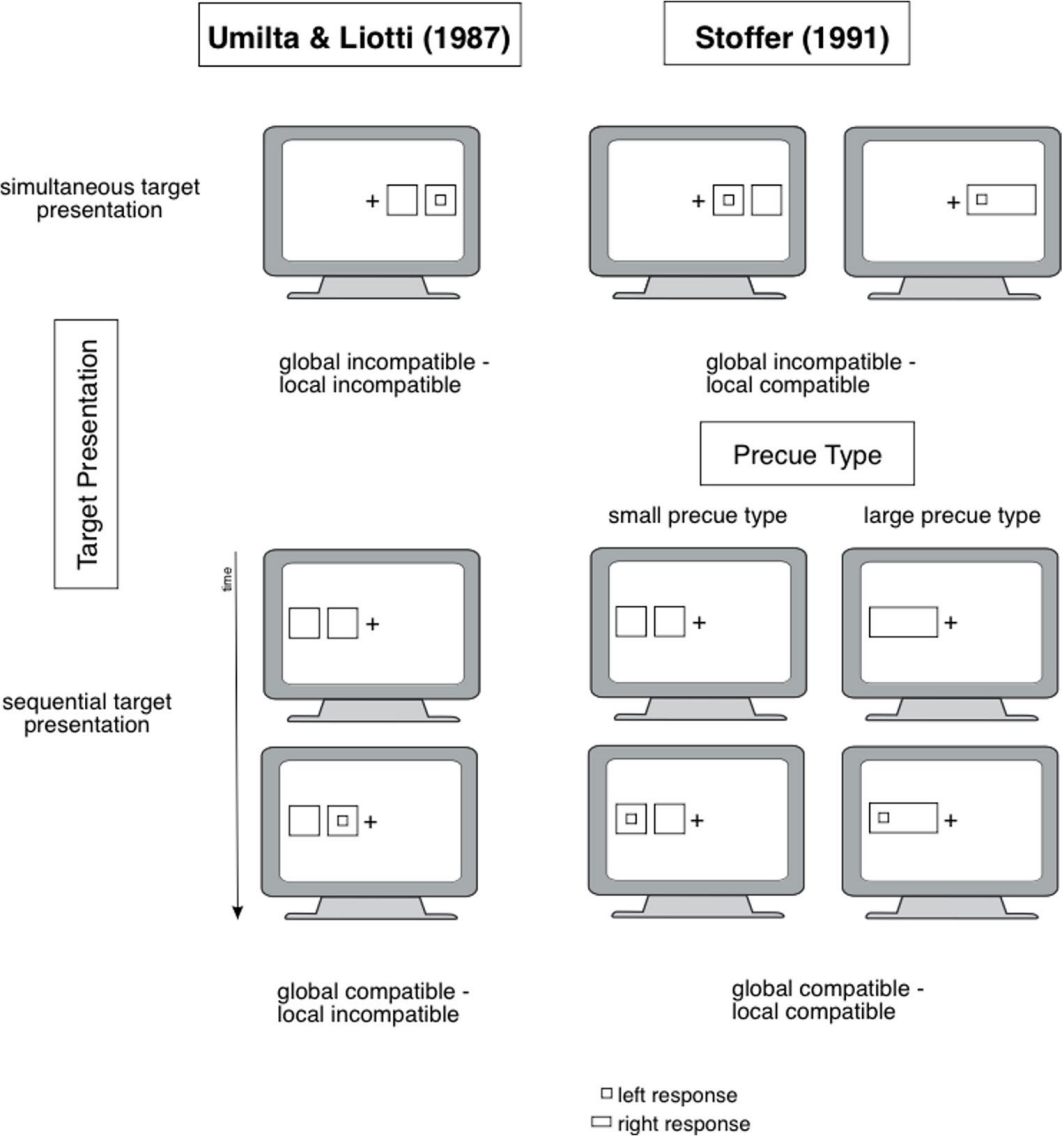
Stimulus display in Umilta & Liotti’s (1987) and Stoffer’s (1991) study. The figure illustrates the stimulus display used by Umilta & Liotti’s (1987, left panel) and Stoffer (1991, right panel). The target was either a square or a rectangle. Two distinct types of Simon effects can be distinguished: a global Simon effect, based on the target’s position relative to the fixation cross (left vs. right side), and a local Simon effect, based on the target’s relative position within each sisde of the screen (left vs. right relative to the local frame). Global or local target positions were varied independently, yielding four trial types: global compatible – local compatible, global compatible – local incompatible, global incompatible – local compatible and global incompatible – local incompatible trials (for left-response square targets). Two modes of target presentation were employed, simultaneous presentation with the precue (top row), or sequential presentation following the precue (bottom row). Umilta & Liotti (1987) used only two small boxes as precue, whereas Stoffer (1991) additionally included one large box and compared different precue types.

We begin by reviewing Umilta &Liotti’s (1987) fundamental study, which inspired the attentional shifting account by Stoffer (1991) and the referential-coding account by Hommel (1993). In the study by Umilta & Liotti (1987), the concept of two distinct types of Simon effects was introduced (see also Fig. 1): one related to the global position of the stimulus on the screen (left vs. right side) and another related to the local position of the stimulus within the two boxes (the relative position within each side). In order to achieve this, a precue (two small boxes) and stimulus (a rectangle or square within one of the boxes) were presented either on the left or right side of the screen’s center. Two presentation conditions were utilized: (1) simultaneous presentation of precue^1^ and stimulus, or (2) sequential presentation, where the precue appeared first, followed after a delay by the stimulus.

In the simultaneous presentation condition, neither Simon effect was observed. However, in the sequential presentation condition, only the Simon effect based on the local, relative position emerged. Umilta & Liotti (Experiment 3 1987) suggested that the formation of two different spatial codes based on the two reference frames results in mutual cancellation of these codes for simultaneous presentation, which leads to the absence of any measurable Simon effect (see also, Umilta & Nicoletti, 1990). For the sequential presentation condition, either Simon effect occurred depending on the experimental manipulations with advanced spatial cueing of the global or local upcoming stimulus’ side. Thus, they reasoned that both global and local spatial codes were in principle formed under the simultaneous presentation condition, yet the outcome of the two independent coding processes was the absence of any Simon effect. Only with sequential presentation, the global or local stimulus code becomes available as a source for the Simon effect depending on the precise experimental manipulations.

Based on these findings, Stoffer (1991) developed an alternative explanation based on differential attentional zooming and focusing processes. Stoffer (1991) suggested that multiple subprocesses of attentional orientation work together to produce spatial codes as indicated by the local Simon effect in Umilta & Liotti’s (1987) study. Stoffer assumed three different attentional subprocesses required in the sequential presentation condition, but not in the simultaneous presentation condition, due to the lack of a potential attention shift. According to Stoffer, the Simon effect arises from the processes of zooming out, shifting attention horizontally, and zooming in (refocussing). These steps can only take place under the sequential presentation condition. In the sequential presentation condition, the first step is an attentional shift from the fixation at the middle of the screen to the precue. Where the attention rests at this stage depends on the precue that is used. If the small boxes are the precue, then attention is shifted to the midpoint between the two boxes. Once the target is presented within one of the two small boxes, a final horizontal attentional shift takes place, from the midpoint between the two small boxes to the target. This final horizontal shift then produces a spatial code which can lead to a Simon effect.

Alternatively, if the large box as precue is used, then the first step involves a horizontal shift from the fixation in the middle of the screen to the precue. The attention is then shifted outwards to encompass the entire precue, rather than simply a midpoint. When a target is finally presented within the precue, the final step is an attentional zooming in, from the precue to the target within the precue. This zooming in does not produce a spatial code, and, therefore, no Simon effect is observed in this condition.

To test his suggestion, Stoffer (1991) conducted an experiment using the same stimulus setup as Umilta & Liotti (1987, Exp. 3), but introduced two different precue types (see also Fig. 1): (1) a small precue consisting of two boxes, as in the original study, and (2) a single large rectangle surrounding the entire area of the two small boxes. The target stimulus (a square or rectangle) was presented either simultaneously within one of the precues or sequentially, where the target appeared after a delay following the precue.

The central test of Stoffer’s (1991) attentional account focused on the differences between the two precue types, the two small, discrete boxes versus the single large box. He reasoned that a Simon effect will only be observed if a horizontal shift in attention with zooming in is the final step in the process of refocussing, which is only possible when two small boxes as precues are used. In contrary, when a large box is presented as precue, the attention focus is more broadly distributed within the single large box which prohibits the last step of refocussing (also labelled zooming in) the target object within the large box to occur. Therefore, Stoffer (1991) expected a local Simon effect for the two small boxes but not for the large box as precue (see also Table 1 for more detailed predictions based on Stoffer’s account). To sum up, when the precue and target stimulus appear simultaneously or when a large box is used as precue, these attentional subprocesses are somewhat disrupted or omitted, leading to an absence of the local Simon effect.

**Table 1:** Theoretical predictions and empirical observations regarding the emergence of Simon effects based on the attentional zooming account (Stoffer, 1991)

Precue			Theoretical predictions	Empirical observations		
	Target presentation	Simon effect Type		Stoffer (1991)	Weeks et al. (1996)	Current study
Small boxes	Simultaneous	Local	- p.129/right column	-?	-?	+
		Global	+ p.129/right column	-	-	+
	Sequential	Local	+ p. 130/left column	+	+	+
		Global	-^1^ p. 130/left column	-	-	+
Large box	Simultaneous	Local	-^2^	-	-?	-
		Global	+^2^	-	-	+
	Sequential	Local	- p. 130/left column	-	+	+
		Global	-^1^	-	-	+

Note: Shown are the theoretical predictions and empirical observations concerning the eight Simon effects in Stoffer’s (1991) as well as the current experiment. Page numbers refer to the Stoffer’s (1991) paper

For the theoretical predictions, a “+” indicates we interpret the theory as predicting the corresponding Simon effect, while a “-“ indicates we interpret the theory as predicting no Simon effect. In order to justify our judgement, we also provide the corresponding text passages in Stoffer’s (1991) study

^1^ According to Stoffer (1991) zooming in is the last step. Thus, the position of the lateral attention shift becomes the new neutral point of the spatial reference frame which is the source for the spatial coding during the last process of refocusing (p. 130, left column). Thus, this overwrites the previous neutral point at the fixation center crucial for the global Simon effect

^2^ There is no explicit statement regarding the simultaneous target presentation condition under a large box as precue. However, we assume that the same would hold true as postulated for the small box condition

For the empirical observations, a “+” indicates a (significant) reported Simon effect, while a “-“ indicates that no significant Simon effect was reported. A “?” indicates that it is unclear from the report whether the Simon effect was substantial or not

These ideas were put to test in Stoffer (1991)’s study which found empirical support for his attentional account. A local Simon effect – based on the target’s relative position within the precue – for the small precue emerged only when the precue and target were presented sequentially. In contrast, for simultaneous presentation of precue and target, the local Simon effect was absent or “negligibly small”^2^ (Stoffer, 1991, p. 131). This pattern aligns with Umilta and Liotti (1987) failure to observe any Simon effect under simultaneous presentation conditions. When using a large box precue, Stoffer (1991) did not observe a reliable local Simon effect based on relative position, regardless of whether the precue and target were presented simultaneously or sequentially. This finding supports Stoffer’s account of differential attentional subprocesses, suggesting that these subprocesses are less effective or cannot occur with the large rectangle due to the absence of a clear reference point needed for attentional refocusing and the distributed nature of attention, unlike for the small precues. According to Stoffer (1991), this pattern of a local Simon effect under sequential target presentation for the small boxes but not for the large box precue confirms the last step of his attentional zooming account, namely the refocusing in order to extract the task-relevant stimulus feature from a non-neutral point between the two small boxes. Consistent with Umilta & Liotti (1987) findings, no global Simon effects — based on the target’s position relative to the screen’s side – were detected under any condition, irrespective of presentation or precue type. Neither Umilta and Liotti (1987) nor Stoffer (1991) explained the absence of this Simon effect in greater detail.

A first attempt to replicate Stoffer’s (1991) study was conducted by Weeks et al. (1996). They used the same stimulus sizes and trial sequence as in the original study: however, several minor deviations were present. These included differences in: (a) the number of trials per block and total trials collected (960 trials for Stoffer, 1991, vs. 800 trials for Weeks et al., 1996); (b) possibly the viewing distance to the screen (50 cm for Stoffer, 1991, vs. not reported by Weeks et al., 1996); (c) the use of a chin rest (used in Stoffer, 1991, but not in Weeks et al., 1996), (d) target-response mapping (fixed in Stoffer, 1991, vs. counterbalanced in Weeks et al., 1996); and (e) upper response time outlier criteria (1200 ms in Stoffer, 1991, vs. 1000 ms in Weeks et al., 1996).

Weeks et al. (1996) found reliable local Simon effects based on the relative position for both the large and small precue types (see also Appendix Table A1 for a full result report). However, unlike Stoffer (1991), they observed no significant difference in the size of the local Simon effect between the two precue types. Additionally, local Simon effects were larger when the target was presented sequentially compared to simultaneous presentation of the target, however it is not clear whether the local Simon effect was present under simultaneous target presentation for both precue types or not. Weeks et al. (1996) concluded that the participants are able to parse either cue type into a left-right spatial code explaining the local Simon effects for large and small precue types. Moreover, the local Simon effects were enhanced for sequential target presentation under any precue type which the authors explained as additional time provided for facilitating stimulus identification. Additional explanations were not given, aside from stating that Stoffer’s attentional shifting account might not be reliable.

Consistent with previous studies (Stoffer, 1991; Umilta & Liotti, 1987), no global Simon effect based on the target’s position relative to the screen side was found.

Alternatives to Stoffer’s attentional zooming and shifting explanation (1991; see also Stoffer, 1993; Stoffer & Yankin, 1994) have also been proposed. Hommel (1993b) introduced the referential coding framework to explain the emergence of Simon effects. Hommel (1993b) argued that the absence of the Simon effect in Stoffer’s (1991) large rectangle precue type can be attributed to the lack of a reference object necessary for forming spatial codes as there is no central object that could have been selected for referential coding of left and right stimuli. This is different for the two small box condition, where each box serves as reference object for the other one containing the target. With this rational, Hommel (1993b) exclusively focused on Stoffer’s large box condition under sequential target presentation by placing a neutral reference object within the large single box. However, the study design did not allow a direct comparison with Stoffer’s study (1991) as the large box condition always contained a reference object next to the target and was never presented without any as in Stoffer (1991). Furthermore, all trials relied on sequential precue and target presentation.

Compared to Stoffer (1991); Hommel (1993b, Experiment 1 and 2) only varied the Relative Position of the target in a centrally presented large box while using stimulus color (and not shape as in Stoffer, 1991) as task-relevant feature for the Simon task. With this setup, Hommel (1993b) reasoned that a local Simon effect should also occur in the large box when a reference object is added, which would serve as evidence against Stoffer’s attentional zooming and shifting account (1991). In line with this prediction, Hommel (1993) demonstrated that when a reference object is included within the large rectangle precue – providing the spatial reference previously lacking – the local Simon effect emerged^3^. In a follow-up experiment, Hommel (1993b, Experiment 3) presented the large box containing the target and reference object on the left or right side of the Screen’s centre – a setup similar to the one of Stoffer (1991). Here, only a local Simon effect emerged, but like for Stoffer (1991) no global one. As Hommel’s (1993b) focus was set on the attentional zooming explanation by Stoffer (1991), he only focused on the sequential target presentation. Therefore, these findings of reliable local Simon effects for the single large box as precue challenged Stoffer’s explanation (1991) based on the feasibility of the attentional refocussing processes for the large box as precue.

To sum up, the question regarding the underlying mechanisms of a Simon effect has been of long-core interest. Generally speaking, two different lines of thought have been proposed, i.e. attention-based (Nicoletti & Umilta, 1989; Rubichi et al., 1997; Stoffer, 1991) and coding-based explanations (Hommel 1993b; Kornblum et al. 1990; Prinz 1990). Differences between these two explanatory lines became evident when explaining the absence of any Simon effect under simultaneous target presentation in Umilta & Liotti (1987)’s study. While the attentional zooming account (Stoffer, 1991) focused on highlighting different attentional subprocesses between simultaneous and sequential target presentation leading to the absence or presence of a local Simon effect, the referential coding account (Hommel 1993b) focused on showing the presence of a local Simon effect for a single large box as precue under sequential target presentation provided the inclusion of a reference object. Interestingly, the original focus of Umilta and Liotti (1987)’s study regarding the emergence of multiple Simon effect was overlooked. Therefore, it is not so surprising that both accounts can explain certain empirical findings regarding the origin of the spatial code generation based on attentional mechanisms or reference coding, but neither account can fully explain the original findings of Umilta & Liotti (1987), nor provide sufficient explanation for the modulations of the Simon effect depending on the presence or absence of a fixation cross or other irrelevant noise stimuli (see Proctor & Lu, 1994). One possible explanation is that diverse findings on the absence or presence of the Simon effect accurately reflect the complexity of the phenomenon, indicating a need for new theoretical developments that integrate the disparate results (as attempted by Proctor & Lu, 1994 or Stoffer & Umilta, 1997). Alternatively, the inconsistencies may arise from limitations in the empirical record itself, which constrains the explanatory power of current theories. The latter issue can be addressed through systematic replications of foundational studies to clarify the robustness and generalizability of earlier findings.

With regard to Stoffer’s (1991) findings, a first replication was provided by Weeks et al. (1996). Despite the strong methodological rigor of Weeks & colleagues (1996), their sample size was similar to that of Stoffer (1991), with only 12 participants compared to 10 in the original study. The reliance on small, potentially underpowered samples remains a well-documented concern in psychological research (Asendorpf et al., 2013; Brysbaert, 2019; Brysbaert & Stevens, 2018; Maxwell, 2004; Vadillo et al., 2016). Given that both studies employed similar Simon tasks but produced divergent results (Stoffer, 1991; Weeks et al., 1996), the evidence remains inconclusive. Either, there could be false negative finding in one or both of the studies, due to limited power. Or, there could be false positive finding due to the larger role of chance associated with smaller sample sizes. Consequently, systematic replications using adequately powered designs are essential to establish a more reliable empirical foundation for theoretical development (Brysbaert, 2019).

**Fig. 2:**
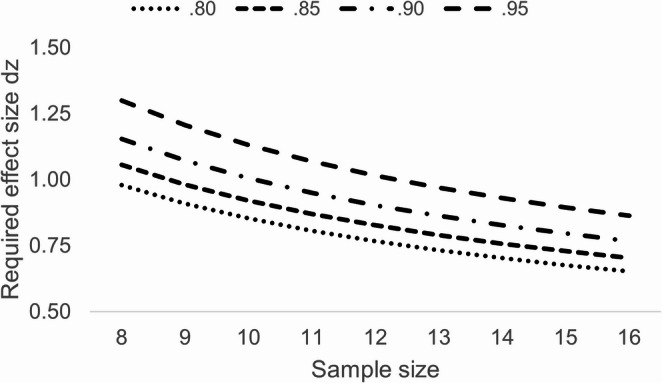
Sensitivity analyses based on the total sample sizes used in previous studies. Output of the sensitivity analysis based on G*Power. Plotted are required effect sizes, *dz*, needed to find a significant results as a function of the power (0.80 to 0.95) and the total sample size for the critical test of a Simon effect, given a *t*-test for dependent means comparing compatible and incompatible stimulus-response mappings

Much of the early research in this area utilized small sample sizes (e.g., Hommel 1993b, *n*_*max*_ = 16; Nicoletti & Umilta, 1989, *n* = 8; Stoffer, 1991, *n*_*max*_ = 12; Umilta & Liotti, 1987, *n* = 12; Weeks et al., 1996, *n* = 12). We conducted a sensitivity analysis using G*Power (Faul et al., 2007), based on sample sizes similar to those in prior studies. The analyses showed that only medium-to-large effect sizes or greater could be detected with at least 80% power (see Fig. 2). This limited power is especially problematic when interpreting null results, such as the absence of a Simon effect. The present paper aims to contribute to the fundamental question of how spatial codes form for task-irrelevant information by providing an adequately-powered replication of a key experiment that initially supported the attentional account underlying the emergence of the Simon effect – Stoffer’s (1991, Experiment 1^4^).

## Present study

As reasoned above, the debate regarding the underlying source of the Simon effect began with the failure to observe a local Simon effect during simultaneous target presentation reported by Umilta and Liotti (1987). Stoffer’s (1991) attentional shifting account was originally proposed to explain the absence of a local Simon effect for simultaneous target presentation in Umilta & Liotti’s (1987) study. His suggestion has sparked considerable debate (Hommel 1993b; Proctor & Lu 1994; Stoffer 1993; Stoffer & Umilta 1997; Weeks et al. 1996). Stoffer (1991) replicated and extended Umilta & Liotti’s study (1987) and likewise found no local Simon effect for simultaneous target presentation. However, in line with his predictions, the local Simon effect reemerged for sequential target presentation with two single boxes as precues, but was absent for a large box precue. Moreover, like Umilta & Liotti’s study (1987), Stoffer (1991) did not find any global Simon effect under any display variant.

Weeks & colleagues (1996) first tried to replicate Stoffer’s results (1991) while including several minor methodological differences compared to Stoffer’s original study (1991), as detailed above. Additionally, Hommel’s (1993b) alternative suggestion of referential coding showed the possibility of a local Simon effect when a reference object was included in the large box precue; however, it did not use the original stimulus display from Stoffer (1991). Moreover, all these studies relied on a relatively small sample size, rendering the evidence for the attentional shifting account as proposed by Stoffer (1991) inconclusive.

To address this, the current study closely replicates Stoffer’s (1991) experiment, following the approach of previous replications (Weeks et al., 1996), but with nearly three times the original sample size (Brandt et al., 2014; Simonsohn, 2015). Participants performed the Simon task over three consecutive days. Each trial involved a large box or two small boxes as precues, presented either simultaneously with the target or sequentially before the target onset.

Based on prior findings (Stoffer, 1991; Umilta & Liotti, 1987), a local Simon effect based on relative position was expected only for two small box precues, and not for large precue. This effect should emerge solely when the two small box precues precedes the target sequentially, but be reduced or absent during simultaneous target presentation. This pattern supports Stoffer’s (1991) attentional zooming account (see Table 1 for a comprehensive overview regarding the emergence of global and local Simon effects based on Stoffer’s (1991) attentional zooming account), whereby zooming in and refocusing occur only with sequentially presented two small boxes as precues, whereas the large box as precue or simultaneous target presentation prevent these attentional subprocesses. Consistent with earlier studies, no global Simon effect based on screen side was anticipated. This prediction relies solely on prior empirical results, yet neither Stoffer’s (1991) attentional zooming account nor Hommel’s (1993b) referential coding account provides a theoretical explanation for this.

With regard to the referential coding account (Hommel 1993b), a local Simon effect is also expected for the large box as precue under sequential target presentation, even without the inclusion of a visible reference object. This was not directly tested in Hommel’s (1993b) study as a reference object was always present within the large box. However, the idea of spatially coding an object in reference to an intentionally defined object suggests that the presence of a local Simon effect is not linked to visibly present reference objects within the large box as precue. Based on Hommel’s (1993b) study, no global Simon effect is expected under any precue type. Since Hommel (1993b) only used sequential target presentation, it is unclear how the target presentation mode influences the local Simon effect. However, based on the another account suggested in parallel (temporal overlap account, Hommel 1993a), it seems reasonable to assume that the local Simon effect should decrease with sequential target presentation given the reduced overlap between task-relevant and task-irrelevant stimulus codes underlying the Simon effect.

In addition, the temporal dynamics of the global and local Simon effect across the reaction time distribution were examined using delta plots (e.g., De Jong et al., 1994; Proctor et al., 2011). Delta plots depict the magnitude of the Simon effect across successive response time bins, ranging from the fastest to the slowest responses. In standard left-right visual Simon tasks, these plots typically reveal a decreasing function (i.e., negative slopes) indicating larger Simon effects for faster compared to slower responses (for overview, Proctor et al., 2011). The mechanisms underlying this decrease remain debated, with accounts attributing it either to passive decay of activation (Hommel 1993a, 1994) or to active suppression processes (Ridderinkhof, 2002). To further inform explanations of the Simon effects within attentional shifting (Stoffer, 1991) and referential coding frameworks (Hommel 1993b), the present distributional analyses examine how the Simon effect is modulated by the experimental manipulation of precue type and target presentation mode. Notably, this approach extends prior work by investigating these modulations in the context of two distinct types of Simon effects.

## Methods

### Subjects

**Fig. 3:**
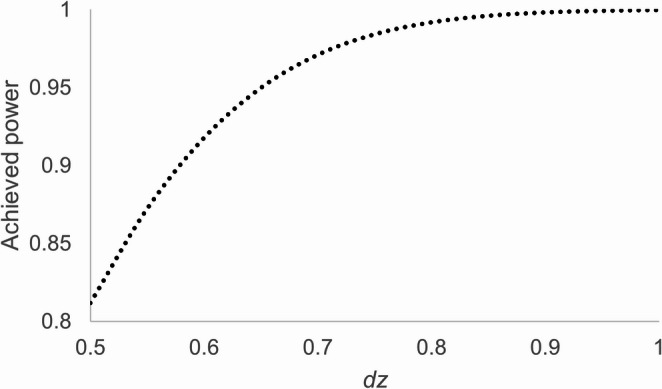
Achieved power of our study. Achieved power as a function of the effect size *dz* for the critical *t*-test for dependent variables comparing the means for compatible and incompatible stimulus-response mappings for our sample of 27 participants

In line with recommendations for replications (Brandt et al., 2014), we aimed to collect at least 2.5 times as many participants as in the original experiment (*N* = 10 participants, Stoffer, 1991). We obtained data from 28 students, with one participant being excluded due to an experimental error. The remaining 27 (20 females, 7 males) had an age range of 20 to 44 years (*M* = 24.38, *SD* = 4.61). With the final sample, power of around 0.80 and more is observed with effect sizes of *d*z ≥ 0.5 (see Fig. 3).

All participants volunteered to take part in the study and provided informed consent prior to participation. All research was conducted in line with the standards laid out in the Declaration of Helsinki (2024), and all experimental protocols were approved by the local ethics committee at the University of Hildesheim (approval number 215). Each participant received partial course credit in return for their participation. All hypotheses, sampling plan, exclusion criteria, and planned data analyses were preregistered before starting data collection (https://osf.io/wj4cf).

### Stimuli and apparatus

All instructions and materials were presented via computer on the screen. Data collection and stimulus presentation were carried out using PsychoPy v202.1.4 presentation software (Peirce et al., 2019) on a Belinea 17-inch CRT monitor.

**Fig. 4:**
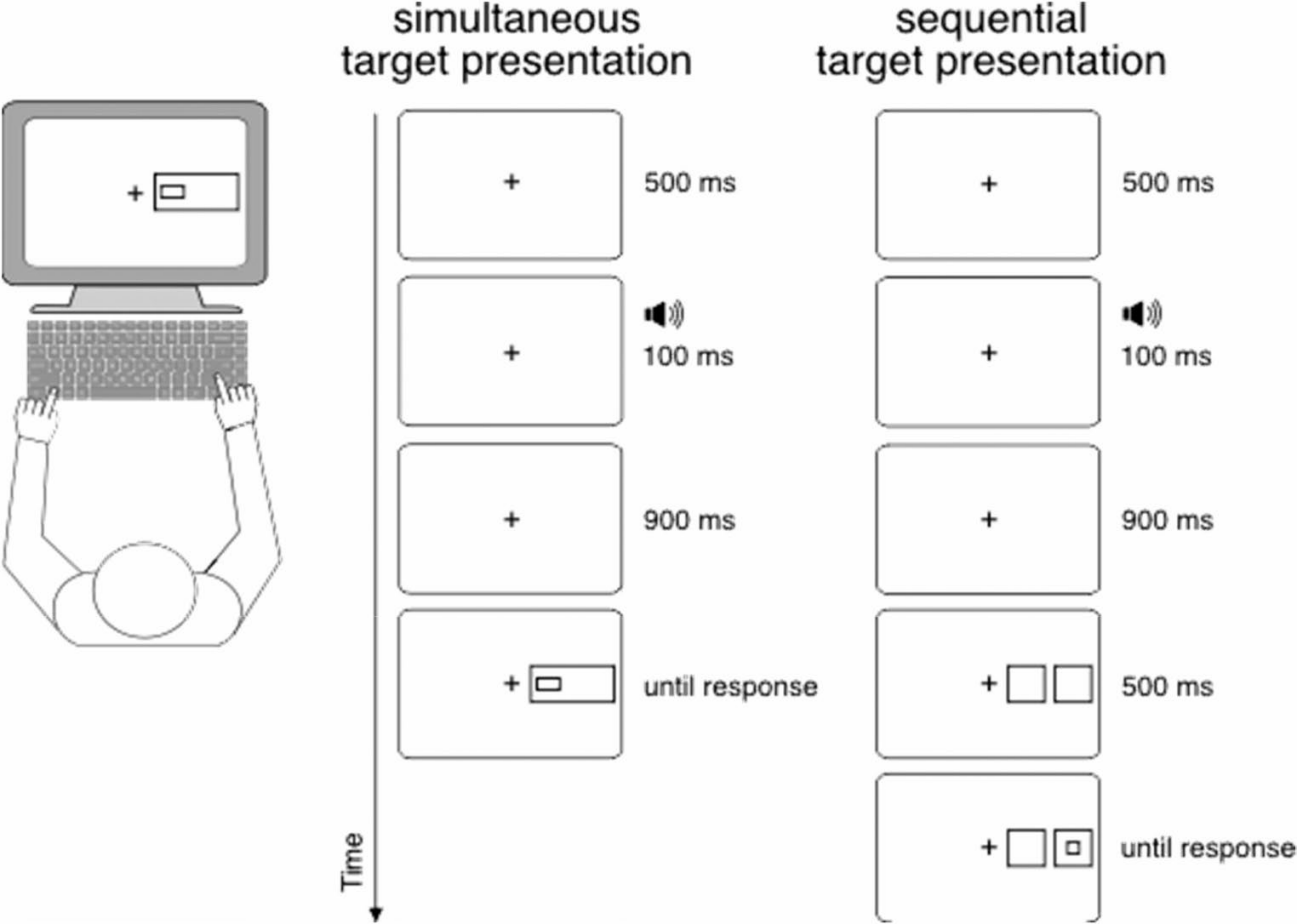
Schematic illustration of the task. A central fixation cross marked the beginning of each trial and remained until a response was made. After 500 ms, a warning tone was presented, which was followed by another blank period. Then one of both precue types was shown (left column: large box; right column: small boxes). The target (left column: rectangle; right column: square) randomly appeared either simultaneously with any of the two precue types (left column) or sequentially, whereby the target followed 500 ms after the display of the precue (right column)

The luminance of the stimuli presented on the monitor was controlled with a lux meter (Gossen, Mavolux 5032c Base). The stimuli (2.82 cd/m²) appeared against a black background with a luminance of around 0.1 cd/m², while the room’s luminance was approximately 30 cd/m². A fixation cross centered on the screen (0.3° × 0.3°) appeared on each trial. As shown in Fig. 4, either two small boxes squares (1.5° x 1.5°) with the center of 3° and 5°, respectively, to the left or right of the fixation cross, or a rectangle (4° x 2°) with the center around 4° to the left or the right of the fixation cross, was used as a precue. A small square of 0.25 ° x 0.25 ° of visual angle in size or a small rectangle of 0.25° x 0.75° served as target stimulus.

### Experimental design

A five-way within-subjects factorial design was employed to examine the Simon effects of Stoffer’s Experiment 1 (Stoffer, 1991). The following within-subjects factors were manipulated: Precue Type (two small boxes vs. one large box), Target Presentation Condition (simultaneous vs. sequential), Screen Position (left vs. right; i.e., target position relative to the screen’s center), Relative Position (left vs. right; i.e., target position relative to the precue), and Response (left vs. right; correct response corresponding to the target, square [left] or rectangle [right]). The key dependent variable was reaction time (RT).

### Procedure

Participants were seated in a quiet room with their head placed in an adjustable chin rest to maintain a constant viewing distance of ~ 50 cm from the screen (see Fig. 4). They placed their forearms on the table and used two keys on a standard computer keyboard placed directly in front of them. Participants were instructed to respond by pressing the “S” key upon seeing a square or the “L” key upon seeing a rectangle with their left or right index fingers, respectively. Participants were instructed to respond as quickly as possible by pressing the correct key, while avoiding errors as much as possible. These keys were separated by 12.5 cm between their centers in a standard German keyboard layout (QWERTZ).

Each trial started with the presentation of a central fixation cross, which remained on the screen till the end of the trial. After 500 ms, a 1000 Hz warning tone (100 ms in duration) was presented through the computer’s loudspeakers, followed by a further interval of 1s. Then one of both precue types (two small boxes or one large box) was shown. The target stimulus (a square or rectangle) occurred randomly at the left or right position of any precue type and remained on the screen until a response was executed.

There were two different target presentation conditions: in the simultaneous target presentation mode, the target occurred simultaneously along with the precue; in the sequential target presentation mode, the target appeared 500 ms after the onset of the precue. Participants classified the target as a square (left response) or a rectangle (right response). Immediate feedback was provided after each trial regarding the accuracy and speed of the response.

There were 192 trials^5^ in each experimental block containing six repetitions of the factorial combination of the following within-subject factors: Precue Type (small boxes vs. large box), Target Presentation (simultaneous vs. sequential), Relative Position (left vs. right), Screen Position (left vs. right), and Response (left vs. right, corresponding with the target), which were all randomly drawn. Participants completed two blocks per session, with a 10-minute break between them. Data was collected over three sessions on three consecutive days, with approximately 24 h between sessions. In total per participant, 1152 trials were collected of which 960 trials were included in the analyses as the first block of the first session served as training and was excluded. Each experimental session lasted about 30 min.

### Data preparation

Only correct responses with a reaction time between 100 ms and below 1,200 ms were included in the analysis^6^. In line with the original study, the first block of the first session was excluded from all analysis. Data preparation was conducted using RStudio v2022.07.1. Fisherian analyses were conducted with SPSS 29.0 as well as RStudio using the packages ezANOVA (Lawrence, 2016) and DMCfun (Ulrich et al., 2015). A total of 5.4% trials were excluded from further analysis since they were identified as outliers (0.2%) or incorrect responses (5.2%). Mean reaction time for correct trials and error rates were analyzed with a 2 (Precue Type: one large box vs. two small boxes) x 2 (Target Presentation: simultaneous vs. sequential) x 2 (Screen Position: left vs. right) x 2 (Relative Position: left vs. right) x 2 (Response: left vs. right) repeated-measures ANOVA. Of central interest are interactions between Relative Position and Response (indicative of a local Simon effect) and Screen Position and Response (indicative of a global Simon effect). Complementary Bayesian statistics were performed using JASP (version 0.18).

Delta plots (DP) were constructed by computing nine RT percentiles (i.e., 10%, 20%, … 90%) for each participant, Target Presentation and Precue Type, separately for the global Screen Position Compatibility and Relative Position Compatibility effects. The corresponding slopes were estimated with a linear fit (cf., Ellinghaus et al., 2024; Mittelstadt & Miller, 2018) and submitted to repeated ANOVAs with the factors Target Presentation Mode, Precue Type and (global or local) Screen or Relative Position Compatibility. In order to investigate potential differences between the slopes of the global and local Simon effect, a repeated measurement ANOVA with the factor Simon Type (global vs. local), Target Presentation and Precue was calculated.

## Results

**Fig. 5:**
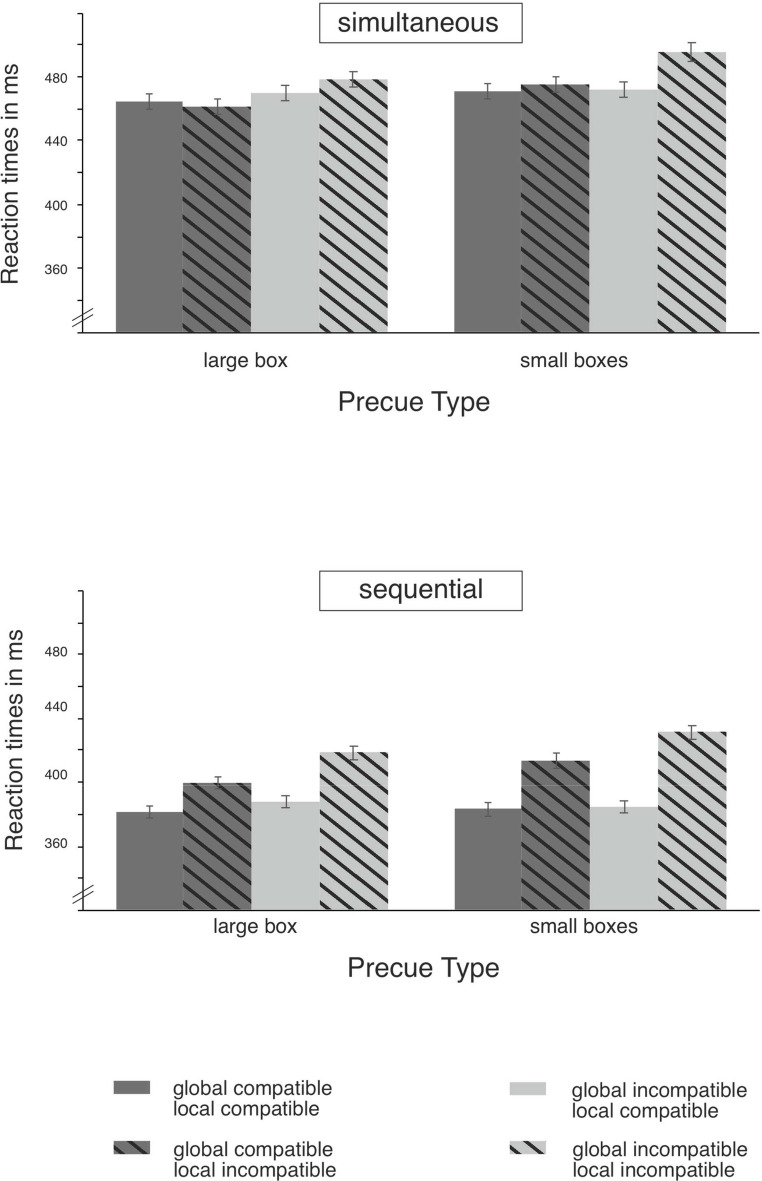
Mean reaction times (with SEM) as a function of precue type (one large box vs. two small boxes) and target presentation (simultaneous vs. sequential) shown separately for global (i.e. the interaction between Screen Position and Response) and local compatibility (i.e. the interaction between Relative Position and Response). Dark grey bars represent globally compatible trials, and light grey bars show globally incompatible trials. Blank bars depict locally compatible trials. Dashed bars show locally incompatible trials

Results are shown in Table 2; Fig. 5. Table 1 in Appendix contains the full summary of the five-factorial ANOVA and offers a comparison with the results reported by Stoffer (1991) and Weeks et al. (1996). Here, we will only focus on the most important results.

**Table 2:** Mean reaction times (in ms with SEM, top part) and error rates (in % with SEM, below part) as a function of cue type, target presentation, screen position, relative position and response

Response times																		
Cue Type: one large box																		
	simultaneous Target presentation							Sequential Target presentation										
	Screen Position							Screen Position										
	Left		Right					Left					Right					
	Relative Position							Relative Position										
Response	Left	Right	Left			Right		Left			Right		Left				Right	
Left	476 ms (± 9)	473 ms (± 10)	475 ms (± 11)			487 ms (± 10)		382 ms (± 8)			404 ms (± 8)		392 ms (± 10)				410 ms (± 9)	
Right	470 ms (± 10)	465 ms (± 11)	450 ms (± 10)			453 ms (± 11)		427 ms (± 8)			384 ms (± 7)		396 ms (± 7)				380 ms (± 8)	
Cue Type: two small boxes																		
	Simultaneous Target presentation							Sequential Target presentation										
	Screen Position							Screen Position										
	Left		Right					Left					Right					
	Relative Position							Relative Position										
Response	Left	Right	Left			Right		Left			Right		Left				Right	
Left	474 ms (± 10)	482 ms (± 11)	469 ms (± 10)			492 ms (± 11)		382 ms (± 9)			409 ms (± 10)		381 ms (± 9)				426 ms (± 10)	
Right	499 ms (± 12)	475 ms (± 11)	469 ms (± 10)			468 ms (± 11)		437 ms (± 8)			389 ms (± 8)		418 ms (± 9)				385 ms (± 9)	
Error rates																		
Cue Type: one large box																		
	simultaneous Target presentation							Sequential Target presentation										
	Screen Position							Screen Position										
	Left		Right					Left					Right					
	Relative Position							Relative Position										
Response	Left	Right	Left			Right		Left			Right		Left				Right	
Left	3.58% (± 0.87)	5.68% (± 1.35)	4.69% (± 1.07)			7.41% (± 1.44)		1.48% (± 0.48)			5.43% (± 1.72)		1.85% (± 0.48)				4.94% (± 1.22)	
Right	4.32% (± 0.73)	4.94% (± 1.16)	3.21% (± 0.97)			3.58% (± 0.87)		11.48% (± 2.18)			3.09% (± 0.75)		7.41% (± 1.59)				3.33% (± 0.85)	
Cue Type: two small boxes																		
	Simultaneous Target presentation							sequential Target presentation										
	Screen Position							Screen Position										
	Left		Right					Left				Right						
	Relative Position							Relative Position										
Response	Left	Right	Left			Right	Left			Right		Left				Right		
Left	2.59% (± 0.7)	6.05% (± 1.36)	3.21% (± 0.88)			6.05% (± 1.61)	1.36% (± 0.45)			5.31% (± 0.88)		1.98% (± 0.65)				8.02% (± 1.61)		
Right	6.91% (± 1.05)	4.44% (± 0.94)	6.42% (± 1.92)			4.57% (± 1.02)	13.95% (± 0.23)			4.44% (± 1.21)		12.47% (± 2.07)				1.73% (± 0.6)

**Table 3:** Global (i.e. based on the screen side) and local (i.e. based on the relative position within one side of the screen) Simon effects (in ms; SEM in parentheses)

Cue Type	Target Presentation	Size of the Simon effect Based on	mean	BF_10_	Interpretation
One large box	Simultaneous	Screen Position	11 ms (± 3)	181.92	Very strong for SE
		Relative Position	3 ms (± 2)	1.14	Anecdotal for SE
	Sequential	Screen Position	13 ms (± 3)	674.70	Very strong for SE
		Relative Position	25 ms (± 3)	192021.35	Very strong for SE
Two small boxes	Simultaneous	Screen Position	11 ms (± 3)	16.40	Strong for SE
		Relative Position	14 ms (± 3)	2299.65	Very strong for SE
	Sequential	Screen Position	10 ms (± 3)	17.83	Strong for SE
		Relative Position	38 ms (± 4)	6120000.00	Very strong for SE

**Fig. 6:**
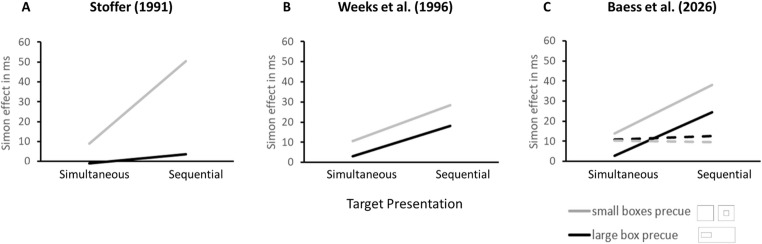
Global and local Simon effects as a function of Precue Type and Target Presentation. This figure was included to allow direct comparison with the results of (**A**) Stoffer (1991), (**B**) Weeks et al. (1996)d the present study (**A**) and (**B**) as both Stoffer (1991) and Weeks et al. (1996) only provided merged data across Screen Position

Firstly, a global and local Simon effect was obtained (see Table 3) with the local Simon effect being larger than the global one. Secondly, the local Simon effect was modulated further by the Target Presentation mode or the Precue Type. The local Simon effect was larger for sequential compared to simultaneous target presentation. Similarly, the local Simon effect was larger for the two small boxes compared to the large box as precue. Thirdly, the five-way interaction with Precue, Target Presentation, Screen Position, Relative Position and Response was significant, whereas the four-way interaction – the central assumption for Stoffer’s (1991) attentional explanation – with Precue, Target Presentation, Relative Position and Response was clearly not. The global and local Simon effects were tested for their robustness under each factorial combination of Precue Type and Target Presentation with single *t*-tests against 0. Local Simon effects were found for all but one condition, *t*_(26)_ ≥ 5.20, *p* ≤.001 (see also Appendix Table A1; Fig. 6): there was no local Simon effect for the large box as a precue with simultaneous target presentation, *t*(26) = 1.58, *p* =.126, which was also observed by Stoffer (1991) and Weeks et al. (1996). The global Simon effects were found for all combinations, *t*_(26)_ ≥ 3.10, *p* ≤.003, which were neither reported by Stoffer (1991) nor by Weeks et al. (1996).

The global and local Simon effects were investigated further with a repeated-measurement ANOVA with the factors Simon Effect Type (global vs. local), Precue Type and Target Presentation. This analysis obtained three main effects, a main effect of Simon Effect Type, *F*_(1,26)_ = 9.34, *p* =.005, *η*_*p*_^*2*^ = 0.264, indicating larger local Simon effects (20 ms ± 2 SEM) than global Simon effects (11 ms ± 2 SEM); a main effect of Precue Type, *F*_(1,26)_ = 15.94, *p* <.001, *η*_*p*_^*2*^ = 0.380, showing larger overall Simon effects for two small boxes (18 ms ± 2 SEM) compared to one large box (13 ms ± 1 SEM) as Precue Type and a main effect of Target Presentation, *F*_(1,26)_ = 34.24, *p* <.001, *η*_*p*_^*2*^ = 0.568, reporting larger overall Simon effects for sequential target presentation (21 ms ± 2 SEM) than simultaneous one (10 ms ± 2 SEM). The interaction between Simon Effect Type and Precue Type was significant, *F*_(1,26)_ = 9.34, *p* =.005, *η*_*p*_^*2*^ = 0.387, reporting larger local Simon effects for two small boxes (26 ms ± 3 SEM) compared one large box as precue (14 ms ± 2 SEM), *t*(26) = 4.84, *p* <.001, as well as compared to the global Simon effect for the two small boxes (10 ms ± 3 SEM), *t*(26) = 4.02, *p* <.001. In contrast, there was no difference between the global (12 ms ± 2 SEM) and local Simon effect (14 ms ± 2 SEM) within the one large box, *t*(26) < 1, *p* =.487, nor a difference between the global Simon effects across two small boxes (10 ms ± 3 SEM) or one large box (12 ms ± 2 SEM) as precue, *t*(26) < 1, *p* =.343.

Moreover, there was an interaction between Simon effect Type and Target Presentation, *F*_(1,26)_ = 25.55, *p* <.001, *η*_*p*_^*2*^ = 0.496, showing a larger local Simon effect for sequential (31 ms ± 4 SEM) than simultaneous (8 ms ± 2 SEM) target presentation, *t*(26) = 7.98, *p* <.001, which was also larger than the global Simon effect under sequential target presentation (11 ms ± 3 SEM), *t*(26) = 4.48, *p* <.001. In contrast, the global Simon effects did not differ between simultaneous (11 ms ± 3 SEM) and sequential target presentation (11 ms ± 3 SEM), *t*(26) < 1, *p* =.902, nor was there a difference between the global and local Simon effect under simultaneous target presentation, *t*(26) < 1, *p* =.368.

### Error rates

The complete output of the five-factorial ANOVA is reported in Appendix Table A1. As done for the response times, we only focus on the most important results here.

In contrast to the reaction times, only a local Simon emerged in the error rates. Similar to the response times, the local Simon effect was modulated by Target Presentation and Precue Type. In line with the response times, the four-way interaction between Precue Type, Target Presentation, Relative Position and Response was not significant, but the five-way interaction with Precue Type, Target Presentation, Screen Position, Relative Position and Response was significant^7^. The global and local Simon effects were tested for their robustness under each factorial combination of Precue Type and Target Presentation with single *t*-tests against 0. Local Simon effects were found for all conditions, *t*(26) ≥ 2.18, *p* ≤.04 – unlike the response time results. Global Simon effects were absent for all but one condition, *t*(26) ≤ 1.35, *p* ≥.199, only the global Simon effect for sequential target presentation for the two small boxes was substantial, *t*(26) ≥ 2.31, *p* =.029.

In addition, an ANOVA was calculated with Simon Type, Precue Type and Target Presentation, which obtained a main effect of Simon Type, *F*_(1,26)_ = 9.73, *p* =.004, $\:{\widehat{\eta\:}}_{G}^{2}$ = 0.08, showing a larger local (3.92% ± 0.66 SEM) than global Simon effect (1.02% ± 0.73 SEM) in accuracy and a main effect of Target Presentation, *F*_(1,26)_ = 20.11, *p* <.001, $\:{\widehat{\eta\:}}_{G}^{2}$ = 0.07, showing less errors for simultaneous (1.11% ± 0.53 SEM) compared to sequential target presentation (3.81% ± 0.65 SEM). Moreover, there were three two-way interactions. First, the interaction of Precue Type and Target Presentation, *F*_(1,26)_ = 7.32, *p* =.012, $\:{\widehat{\eta\:}}_{G}^{2}$ = 0.01, showing larger local Simon effects for two small boxes (4.88% ± 0.77 SEM) compared to one large box (2.96% ± 0.71 SEM) as precue, *t*(26) = 2.87, *p* =.008, and a larger local Simon effect (4.88% ± 0.77 SEM) than global Simon effect (0.83% ± 0.87 SEM) for two small boxes as precue, *t*(26) ≥ 3.61, *p* =.001. Second, the interaction of Precue Type and Simon Type, *F*_(1,26)_ = 6.22, *p* =.019, $\:{\widehat{\eta\:}}_{G}^{2}$ = 0.01, showing larger Simon effects for two small boxes (2.87% ± 0.61 SEM) compared to one large box (2.07% ± 0.54 SEM) as precue. Third, the interaction of Target Presentation and Simon Type, *F*_(1,26)_ = 11.01, *p* =.003, $\:{\widehat{\eta\:}}_{G}^{2}$ = 0.04, showing larger Simon effects for sequential (3.83% ± 0.66 SEM) compared to simultaneous (1.11% ± 0.53 SEM) target presentation.

### DP analysis

**Fig. 7:**
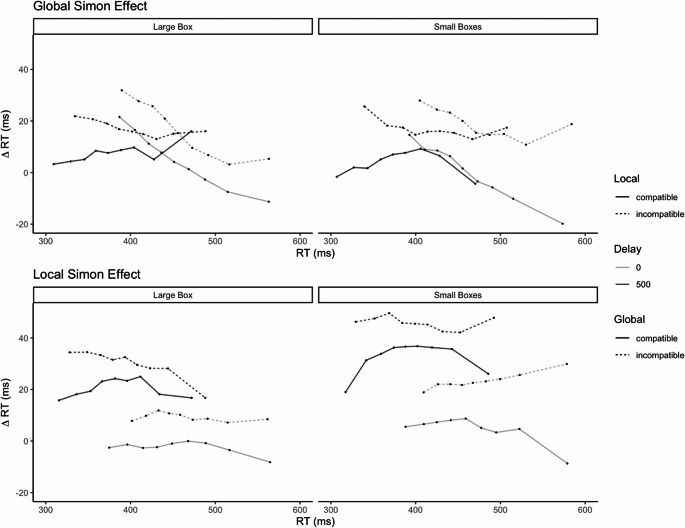
Delta plots for the global (upper part) and local (lower part) Simon effects as a function of Precue Type, Target Presentation and local relative Position (upper part) or global Screen Position (lower part) compatibility

As can be seen in Fig. 7, the DPs for the global and local Simon effects were modulated differentially over time. Whereas the DPs for the global Simon effect follow the typical negative-going trend, the DPs for the local Simon effect appear to stay relatively constant across the RT distribution. Moreover, target presentation mode differently influenced the slopes of the Simon effects.

To statistically assess the interpretation based on visual inspection, we summarized the DP separately for the global Screen Position Compatibility and local Relative Position Compatibility for each participant, Target Presentation, Precue Type and local Relative Position or global Screen Position Compatibility with a linear regression model.

### Global Simon effect

As can be seen in Fig. 7, the DPs for the global Simon effect followed the typical negative-going trend, *M* = −0.081, 95% CI [−0.125, −0.037], *t*(26) = 3.76, *p* <.001, when averaged across all conditions.

The slope was numerically steeper for the simultaneous target presentation (*M* = −0.165, 95% CI −0.214, −0.116]), compared to the sequential one (*M* = 0.003, 95% CI [−0.004, 0.047]), *F*(1,26) = 24.28, *p* <.001, $\:{\widehat{\eta\:}}_{G}^{2}=\:.11$.

### Local Simon effect

As can be seen in Fig. 7, the DPs for the local Simon effects did not follow the typical negative-going trend but stayed relatively constant across the RT distribution, *M* = −0.014, 95% CI [−0.054, 0.026], *t*(26) = −0.735, *p* =.469, showing a non-significant difference of the slope from zero when averaged across conditions. The corresponding ANOVA did not obtain any significant main effects or interactions, all *F*_(1,26)_ ≤ 2.48, *p* ≥.128.

### Slopes of global and local Simon effects

The slopes of the global and local Simon effects were compared with an ANOVA with the factors Simon effect Type, Precue Type and Target Presentation mode. This analysis obtained a main effect of Simon effect Type, *F*_(1,26)_ = 5.99, *p* =.021, $\:{\widehat{\eta\:}}_{G}^{2}$ = 0.04, showing a steeper slope for the global Simon effect (*M* = −0.081, 95% CI [−0.115, −0.046]) than the local Simon effect (*M* = −0.014, 95% CI [−0.044, 0.016]). The slopes differed between simultaneous (*M* = −0.090, 95% CI [−0.123, −0.057] and sequential Target Presentation (*M* = −0.005, 95% CI [−0.036, 0.026]), *F*_(1,26)_ = 11.84, *p* =.002, $\:{\widehat{\eta\:}}_{G}^{2}$ = 0.06, which were further modulated by the Simon effect Type, *F*_(1,26)_ = 15.08, *p* =.001, $\:{\widehat{\eta\:}}_{G}^{2}$ = 0.006. The interaction was dissolved by comparing the slope for the local Simon effect (*M* = −0.014, 95% CI [−0.054, 0.026]) and global Simon effect which differed under simultaneous target presentation (*M* = −0.165, 95% CI [−0.214, −0.116]), *t*(26) = −4.49, *p* <.001, but not under sequential target presentation, *t*(26) < 1, *p* =.463. Moreover, the slopes of the global Simon effect differed between simultaneous (*M* = −0.165, 95% CI [−0.214, −0.116]) and sequential target presentation (*M* = 0.003, 95% CI [−0.040, 0.047]), *t*(26) = 4.93, *p* <.001, but not for local Simon effect (simultaneous: *M* = −0.014, 95% CI [−0.054, 0.026]; sequential: *M* = −0.014, 95% CI [−0.059, 0.031]), *t*(26) < 1, *p* =.979. (Fig. 7).

## Discussion

The present experiment aimed to directly replicate Stoffer’s (1991) study, which extended the earlier work of Umilta & Liotti (1987). Stoffer (1991) investigated both global and local Simon effects under simultaneous and sequential target presentation, using two distinct reference objects as external precues: a large box and two small boxes. Consistent with Umilta & Liotti’s findings (1987), Stoffer (1991) observed only a local Simon effect for sequential target presentation when two small boxes were employed as reference objects, whereas no (local or global) Simon occurred for the large box as reference object. Weeks et al. (1996) previously failed to replicate these findings. Both studies used small sample sizes while obtaining discrepant results. Therefore, the empirical evidence from these studies remains inconclusive.

In contrast, the present study offers adequately-powered empirical evidence regarding the role of attentional mechanisms underlying the Simon effect. Crucially, our study demonstrated (i) that the local Simon effect was modulated by the type of the reference objects (two small boxes or one large box) and target presentation condition, and (ii) that reliable global *and* local Simon effects emerged under both simultaneous and sequential target presentation mode. To better understand these patterns, we first explore how our results are consistent with and how they deviate from previous research findings. This is followed by a discussion of both accounts in explaining the current findings.

Previous studies (Stoffer, 1991; Weeks et al., 1996) as well as the current research showed two consistent findings (see also Tables 1 and 3): (i) the local Simon effect was larger under sequential target presentation than under simultaneous one and (ii) the absence of a local Simon effect under simultaneous target presentation for the large box as precue. Stoffer (1991) showed that both, the type of precue reference objects and the target presentation mode interacted to influence the magnitude of the local Simon effect. In particular, Stoffer found the largest local Simon effect when two small boxes preceded the target sequentially, but a “negligibly small effect (9 ms)” under simultaneous presentation (Stoffer, 1991, p. 131). Additionally, no local Simon effect was found when one large box was used as reference object – regardless of target presentation mode. Weeks et al. (1996) similarly reported an increase in the local Simon effect for sequential versus simultaneous target presentation, although, contrasting with Stoffer, they found this effect for both precue types. Likewise, our study showed an increase of the local Simon effect for sequential compared to simultaneous target presentation, which was not limited to the two small boxes as precue. Weeks et al. (1996) and our study showed that this modulation also occurs with a large box as precue. In this point, Weeks et al. (1996) and our study differ from Stoffer’s (1991) original findings. Stoffer (1991) found the modulation of the local Simon effect by target presentation mode only when two small boxes served as precues. In addition, our study revealed generally larger local Simon effects for two small boxes as precue than for one large box, a pattern that was not reported by Weeks et al. (1996), but mirrored Stoffer’s findings (Stoffer, 1991).

In sum, Stoffer (1991) observed a reliable modulation of the local Simon effect under the sequential target presentation only for the two small boxes as precue (i.e., a four-way interaction between precue type, target presentation mode, relative stimulus position, and response), describing both, target presentation and precue type as mutually influencing factors for the local Simon effect. In contrast, the replications by Weeks et al. (1996) and our current study found no evidence for the reported mutual dependency. Instead, Weeks et al. (1996) showed that target presentation (simultaneous or sequential) alone influenced the local Simon effect in a similar vein in both precue types (as shown by the three-way interaction between target presentation mode, relative stimulus position and response). Our current study replicated this finding of the dependency of the local Simon effect on target presentation. Additionally, our research showed that small boxes as precue increased the local Simon effects (as shown by the three-way interaction between precue type, relative stimulus position and response), yet both influencing factors were independent from one another (as shown by the absence of the four-way interaction between precue type, target presentation, relative stimulus position and response). Our replication study demonstrated that the local Simon effect was modulated by both precue type (i.e., two small boxes or one large box) and the target presentation mode (i.e., simultaneous or sequential).

Strikingly, across all studies, including ours, no reliable local Simon effect was found for the one large box as precue reference object with simultaneous target presentation mode; any effect observed was very small and not statistically significant^8^. This finding has now been replicated throughout all studies (cf. Fig. 6), increasing the need for a sufficient explanation of the absence of the local Simon effect within the large box as precue. According to Stoffer’s attentional zooming explanation (1991), no local Simon effect under simultaneous target presentation should occur due to the lack of time to shift the attentional focus from screen’s center. This explanation would hold for both the two single boxes or the one large box as precue. However, the empirical records show that the local Simon effect is only absent for the one large box, but not for the two small boxes as precue under simultaneous target presentation.

In contrast, the referential coding account (Hommel 1993b) was motivated by the sequential target presentation, but did not test simultaneous target presentation. Hommel (1993b) showed that the large box as precue is well suited to foster spatial codes based on the relative position provided a visible reference object was included. Weeks et al. (1996) as well as the current study extended this by reporting reliable local Simon effect for the one large box under sequential target presentation even without a visible reference object given. However, the referential coding account (Hommel 1993b) fails to explain the absence of a local Simon effect for the one large box under simultaneous target presentation while the local Simon effect is present for the two small boxes.

Taken together, both accounts have difficulties in explaining the selective absence of the local Simon effect under simultaneous target presentation for the one large box. Moreover, both accounts fail to explain the other central finding of our current study: substantial global Simon effects under all variants of precue type and target presentation.

Our findings reveal substantial global Simon effects in the response times (but not in accuracy data) for both two small boxes and one large box, under both simultaneous and sequential target presentation, which was never observed in previous studies using this stimulus display (Stoffer, 1991; Weeks et al., 1996) nor under the sequential target presentation (Hommel 1993b). Importantly, the global Simon effect was not modulated by either precue type or the target presentation mode, neither in the response times, nor in the slope analysis.

### Multiple Simon effects based on different reference frames

Substantial global and local Simon effects — reflecting the activation of two distinct spatial reference frames — occurred concurrently across both simultaneous and sequential target presentation. This contrasts with earlier reports by Stoffer (1991) and Weeks et al. (1996), where concurrent global and local effects were absent. Even Umilta & Liotti’s study (1987) or Hommel (1993b) found only a local Simon effect for sequential target presentation, despite the visual display allowing for global and local Simon effects. None of these studies reported global and local Simon Effects emerging together.

Furthermore, when global and local Simon effects occurred concurrently in other studies, it was exclusively under sequential target presentation (Roswarski & Proctor, 1996) or when spatially informative cues about the target’s screen position were provided (Lamberts et al., 1992). Lamberts et al. (1992) expanded on the small-box stimulus arrangement used in earlier studies (see Stoffer, 1991; Umilta & Liotti, 1987) by incorporating eight possible spatial target locations (four on each side of the screen). Although the boxes were presented simultaneously with the targets, participants were given a lateralized fixation cross indicating the side of the upcoming target. This design led to three distinct Simon effects: a global effect based on the target’s position relative to the screen center, a hemifield effect based on the target’s position within one screen side, and a local effect based on the target’s position relative to hemifield side.

Roswarski & Proctor (1996) examined how different task-relevant stimulus features – color classification (red vs. green), easy shape classification (circle vs. rectangle), and difficult shape classification (square vs. rectangle) – affect the Simon effects, using stimulus layouts from Umilta and Liotti (1987) (Experiment 2) or Lamberts et al. (1992) (Experiment 1). Replicating Lamberts et al.’s design, they found three Simon effects (global, hemifield, and local) only in the color-classification task. For easy shape-classification, two Simon effects (hemifield and local) emerged, while only the global Simon effect appeared for difficult shape-classification. In their second experiment, which used simultaneous target presentation with the Umilta and Liotti stimulus arrangement, only the global Simon effect occurred for both color and easy shape-classification. This global Simon effect contrasted with Umilta and Liotti’s (1987) original report, where no (global or local) Simon Effects were found under simultaneous target presentation. In a third experiment, Roswarski and Proctor (1996) introduced a modified stimulus layout with four possible target positions along the horizontal plane, delineated by three vertical lines. In this setup, both global and local Simon effects appeared for all stimulus classifications under sequential target presentation.

Taken together, previous studies did not provide evidence for multiple concurrent Simon effects — based on different spatial reference frames — unless sequential target presentation was involved. However, recent research using a different approach detected both global and local Simon effects even without explicit information about the target’s position or distinct reference objects (Baess & Bermeitinger, 2025; Baess et al., 2023; Wang et al., 2016).

Wang & colleagues (2016) had participants respond to the color of a fork placed at various locations on a plate, which itself was positioned on either the right or left side of the screen. Baess & colleagues (2023, 2025) used a similar design in which participants responded to the color of a ball held in either hand of a stick-figure manikin that could appear on either side of the screen center. Both procedures activated two spatial reference frames concurrently: a global frame relating the stimulus position to the screen center, and a local frame relating the stimulus position relative to the reference object (the fork on the plate or ball in the manikin’s hand). Notably, Wang et al. (2016) observed only concurrent global and local Simon effects when the relative position on the fork was reinforced in a forerunner task.

Our studies (Baess & Bermeitinger, 2025; Baess et al., 2023) provided evidence for concurrent global and local Simon effects, indicating two active reference frames. Crucially, in both cases, the target stimulus was embedded within the same reference object (cf. Baess et al., 2018), differing from previous research that used external boxes as reference objects (Lamberts et al., 1992; Roswarski & Proctor, 1996; Stoffer, 1991; Umilta & Liotti, 1987). Interestingly, Wang et al. (2016) found that the global and local Simon effects were similar in magnitude whereas our studies showed that the global Simon effect tended to be larger (Baess & Bermeitinger, 2025). This differential size possibly reflects the weighting of spatial coding processes, where global spatial features linked to the broader context or environment tend to dominate over local object-centered coding (Baess et al., 2023).

However, our replication demonstrates that the local Simon effect can be larger than the global Simon effect when using external reference objects. Multiple Simon effects can occur concurrently for both the same object and for external references objects – even with simultaneous target presentation. This challenges previous implicit assumptions (cf. Umilta & Liotti, 1987) that sequential target presentation with artificially partitioning of target and the reference objects (see Lamberts et al., 1992; Roswarski & Proctor, 1996; Stoffer, 1991; Umilta & Liotti, 1987) are necessary for multiple concurrent Simon effects. Hence, our findings suggest that such sequential target presentation is not required to elicit multiple Simon effects, offering a fresh perspective on the spatial coding mechanisms underlying these effects.

The presence of global and local Simon effects across different precues and target presentation modes together with the weak associations between effect sizes, suggests a partial dissociation between the mechanisms underlying global and local Simon effects. Indeed, the overall global and local Simon effects in our study were not significantly correlated^9^ (*r*(27)= 0.15, *p* =.455), and only a marginal correlation emerged between the two effects for simultaneous target presentation within the two small boxes as precue (*r*(27) = 0.407, *p* =.035), whereas all other correlations between global and local Simon effects within each precue and presentation condition were clearly small. Further evidence comes from the slope differences in delta plots showing the response time distribution. Whereas the global Simon effects follows the typical decreasing slope pattern with smaller Simon effects for slower reaction time bins (for overview, Dittrich et al., 2014; Pratte et al., 2010; Proctor et al., 2011), the local Simon effect follows a different pattern and remains similar in size across the temporal reaction time distribution. Thus, these findings provide initial evidence that global and local Simon effects may be governed, at least in part, by distinct cognitive mechanisms (see for a similar argument, Mittelstädt & Miller, 2020).

## Conclusion

Taken together, these empirical findings invite a reconsideration of existing theoretical models to better account for the concurrent presence and differential modulation of global and local Simon effects.

Two major accounts have been proposed to explain the origin of spatial codes underlying the Simon effect: the attentional zooming account (Stoffer, 1991) and the referential coding account (Hommel 1993b). Both the attentional zooming and referential coding accounts rigorously explain the origins of spatial codes underlying the Simon effects, though from somewhat mutual exclusive perspectives that emphasize attentional or object-based reference frames, respectively. Our findings provide a novel perspective on the mechanisms underlying the Simon effect: the hybrid spatial coding account of Simon effects. Prior studies often reported either local or global Simon effects when developing the attentional zooming (Stoffer, 1991; Umilta & Liotti, 1987) or referential coding accounts (Hommel 1993b), possibly due to small sample sizes limiting global Simon effect detection or stimulus designs emphasizing only local spatial coding. The concurrent presence of both, i.e. global and local, Simon effects in our study, along with their selective modulation by precue type and target presentation, supports distinct underlying mechanisms.

According the hybrid spatial coding account, the global Simon effect, unaffected by these factors, likely reflects automatic attentional capture towards the target’s global screen position. This explains well why a global Simon effect was observed under simultaneous and sequential target presentation for small boxes and one large box as precue in the current study. Therefore, the global Simon effect is apparently linked to any changes in the spatial location away from the neutral screen’s center supporting generally a broad focus. In contrast, the local Simon effect’s sensitivity to reference objects and presentation timing implicates referential coding of relative position. Here, the local Simon effect supports a narrower and more detailed focus allowing the spatial codes based on the relative position within either side of the screen’s center. This explains well why the local Simon was found under sequential target presentation and simultaneous target presentation with the exception of the one large box as precue and why different precue types modulated its size. Referential coding mechanisms may change depending on the saliency of the reference objects. This implies differential processing weights for reference frames depending on their saliency. Thus, a single large box as precue may differ in saliency to two small boxes as precue, which potentially results in the absence of a local Simon effect when the reference coding mechanisms could not benefit from the sequential target presentation. Future research should continue to investigate how these mechanisms interact and are differently engaged under varying perceptual and environmental conditions (see also, Baess & Bermeitinger, 2025; Baess et al., 2023).

To sum up, our research demonstrates the concurrent presence of both global and local Simon effects in conditions in which previous studies failed to observe both effects simultaneously (Stoffer, 1991; Umilta & Liotti, 1987; Weeks et al., 1996). Importantly, only the local Simon effect was modulated by the type of reference objects or target presentation mode, highlighting distinct mechanisms underlying the global and local Simon effects. This suggests a hybrid explanation involving attentional focusing and referential coding processes. Understanding the dynamic interplay between these multiple concurrent Simon effects may offer important theoretical advances, providing a coherent explanatory framework for diverse empirical findings in spatial cognition and response selection.

## Supplementary Information

Below is the link to the electronic supplementary material.

Supplementary file 1 (DOCX 18.9 KB)

## Data Availability

The data that support the findings presented in this paper are openly available on Open Science Framework: [osf.io/54pq6](https:/osf.io/54pq6). Both experiments were preregistered on the Open Science Framework: osf.io/wj4cf.

## Appendix

**Table 4:** Overview of the ANOVA results obtained for the repeated measurement ANOVA with the factors Precue Type, Target Presentation, Screen Position, Relative Position and Response. Additional columns show the results reported by Stoffer (1991) and Weeks at al. (1996)

	Stoffer (1991)	Weeks et al. (1996)		Present Study	
			Response times		Error rates
Main effect Precue	As such not reported under Exp 1, however, based on Exp 2 (p.133), one might conclude that there was a main effect	*F*_(1,11)_ = 30.71, *MS*_*e*_ = 214.40, *p* <.001: faster responses for the large box (399 ms) compared to two small boxes (407 ms)	*F*_(1,26)_ = 43.31, *p* <.001: faster responses for the large box (433 ms ± 8 SEM) compared to two small boxes (441 ms ± 9 SEM)		*F*_(1,26)_ = 5.65, *p* =.025, less errors for large box (4.78% ± 0.76 SEM) (compared to two small boxes 5.59% ± 0.78 SEM)
Main effect Target Presentation	*F*_(1,9)_ = 414.99, *p* <.0001: sequential presentation faster (418 ms) compared to sequential target presentation (523 ms)	*F*_(1,11)_ = 282.11, *MS*_*e*_ = 2182.36, *p* <.001: sequential presentation (363 ms) faster compared to stimultaneous target presentation (443 ms)	*F*_(1,26)_ = 582.96, *p* <.001: faster responses for sequential (400 ms ± 8 SEM) compared to simultaneous target presentation (474 ms ± 10 SEM)		Not significant, *F*_(1,26)_ = 2.19, *p* =.151
Main effect Screen Position,			*F*_(1,26)_ = 11.67, *p* =.002: faster responses for stimuli on the right side (434 ms ± 9 SEM) compared to the left side of the screen (439 ms ± 8 SEM)		Not significant, *F*_(1,26)_ < 1, *p* =.490
Main effect Response			Not significant, *F*_(1,26)_ < 1, *p* =.476		*F*_(1,26)_ = 14.46, *p* <.001: less errors with left response (4.35% ± 0.71 SEM) compared to right response (6.02% ± 0.86 SEM)
Interaction Screen Position x Relative Position	*F*_(1,9)_ = 31.51, *p* <.001; slower responses for more eccentric stimuli	*F*_(1,11)_ = 99.50, *MS*_*e*_ = 205.78, *p* <.001: faster responses to stimuli presented to right within the cue than to left when cue was presented to right; reversed when cue presented to left	*F*_(1,26)_ = 49.25, *p* <.001: significant differences between all combinations of both factorial levels, *t*_(26)_ ≥ 3.27, *p* ≤.003, except these two comparisons: (i) left side of the screen and left relative position compared to trials on the right side of the screen and right relative position and (ii) left side of the screen and right relative position compared to trials on the right side of the screen and left relative position		Not significant, *F*_(1,26)_ < 1, *p* =.501
Interaction Precue x Target Presentation		*F*_(1,11)_ = 16.04, *MS*_*e*_ = 174.17, *p* <.01; faster responses for large box than small boxes for simultaneous presentation	Not significant, *F*_(1,26)_ = 3.22, *p* =.084		Not significant, *F*_(1,26)_ = 3.6, *p* =.069
Interaction Precue x Relative Position			Not significant, *F*_(1,26)_ < 1, *p* =.656		*F*_(1,26)_ = 4.47, *p* =.044: more errors for left relative position in small boxes (6.11% ± 0.86 SEM) compared to the left relative position in large box (4.75% ± 0.71 SEM), *t*_(26)_ = 2.9, *p* =.008
Interaction Precue x Response			*F*_(1,26)_ = 25.36, *p* <.001: faster responses for the large box precue (428 ms ± 9 SEM) than the small boxes precues (442 ms ± 9 SEM) for the right button only, *t*_(26)_ = 7.79, *p* <.001.		*F*_(1,26)_ = 7.22, *p* =.012: more errors for the small boxes requiring right response (6.87% ± 1.01 SEM) compared to left response (4.32% ± 0.67 SEM), *t*_(26)_ = 3.56, *p* =.001
Interaction Target Presentation x Relative Position		*F*(_1,11)_ = 20.41, *MS*_*e*_ = 115.66, *p* <.01: RTS were faster for left stimulus position compared to right for simultaneous presentation; this was reversed for sequential presentation	*F*_(1,26)_ = 7.04, *p* =.013: significant differences between all combinations of factor levels, *t*_(26)_ ≥ 21.95, *p* <.001, except responses between left and right Relative Position did not differ, neither for the simultaneous nor the sequential target presentation		*F*_(1,26)_ = 19.41, *p* <.001: more errors for left Relative Position for sequential (6.5% ± 0.97SEM) compared to simultaneous target presentation (4.37% ± 0.61 SEM, *t*_(26)_ = 3.51, *p* =.002, and compared to right Relative Position for sequential target presentation (4.54% ± 0.83), *t*_(26)_ = 4.53, *p* <.001
Interaction Target Presentation x Response			*F*_(1,26)_ = 13.73, *p* =.001: significant differences for all factorial combinations, *t*(26) ≥ 11.46, *p* <.001, except there were no differences between the left and right responses, neither for simultaneous nor sequential target presentation		*F*_(1,26)_ = 17.74, *p* <.001: more errors for right button under sequential target (7.24% ± 0.1.11 SEM) compared to simultaneous target presentation (4.8% ± 0.7 SEM), *t*_(26)_ = 3.45, *p* =.002 and compared to left button under sequential target presentation (3.8% ± 0.7 SEM), *t*_(26)_ = 5.77, *p* <.001
Interaction Screen Position x Response (i.e. global Simon effect)			*F*(1,26) = 25.75, *p* <.001: global Simon effect of 11 ms ± 2 SEM		Not significant, *F*_(1,26)_ = 2.90, *p* =.101
Interaction Relative Position x Response (i.e. local Simon effect)		*F*_(1,11)_ = 26.24, *MS*_*e*_ = 856,73, *p* <.001	*F*_(1,26)_ = 72.36, *p* <.001: local Simon effect of 20 ms ± 2 SEM		*F*_(1,26)_ = 34.17, *p* <.001: local Simon effect of 4.01% ± 0.69 SEM
Interaction Precue x Relative Position x Response	Probably significant (p. 131): no Simon effect for each target presentation for the large box, but Simon effect for each target presentation for small boxes	Not significant, *F*_(1,11)_ = 0.526, *MS*_*e*_ = 252.50: no differences in local Simon effect between two small boxes (19.5 ms) and one large box (10.5 ms)	*F*_(1,26)_ = 23.44, *p* <.001: larger local Simon effect for small boxes (26 ms ± 3 SEM) compared to the large box as precue (14 ms ± 2 SEM), *t*_(26)_ = 4.84, *p* <.001		*F*_(1,26)_ = 8.48, *p* =.007, larger local Simon effect in small boxes (5.11% ± 0.84 SEM) compared to large box (2.92% ± 0.72 SEM),*t*_(26)_ = 2.9, *p* =.007
Interaction Target Presentation x Relative Position x Response		*F*_(1,11)_ = 13.96, *MS*_*e*_ = 506,63, *p* =.003: larger local Simon effect (23 ms) for sequential target presentation compared to simultaneous one (7 ms)	*F*_(1,26)_ = 63.65, *p* <.001: larger local Simon effect (31 ms ± 4 SEM) for sequential target presentation compared to the simultaneous one (8 ms ± 2 SEM)		*F*_(1,26)_ = 27.21, *p* <.001: larger local Simon effect in errors for sequential target presentation (6.22% ± 1.01 SEM) compared to simultaneous one (1.81% ± 0.53 SEM), *t*_(26)_ = − 5.26, *p* <.001
Interaction Precue x Target Presentation x Relative Position x Response	*F*_(1,9)_ = 16.85, *p* <.01	Not significant, *F*_(1,11)_ = 0.526, *MS*_*e*_ = 261.63	Not significant, *F*_(1,26)_ = 0.281, *p* =.601		Not significant, *F*_(1,26)_ = 3.02, *p* =.094
Interaction Precue x Target Presentation x Screen Position x Relative Position x Response			*F*_(1,26)_ = 11.48, *p* =.002		*F*_(1,26)_ = 4.87, *p* =.036

Empty cells mean that no statistics was reported for this influence. We also considered the discussion sections for this judgement. Error rates were not reported in Stoffer (1991) and Weeks et al. (1996)

## Footnotes

1 Note that the term “precue” can be somewhat misleading, as the cue does not always precede the stimulus. Nevertheless, the term is retained here for consistency with prior research.

2 It should be noted that Stoffer (1991) did not report inferential statistics for the local Simon effect, leaving some ambiguity about whether it was truly absent or merely reduced compared to the sequential presentation of precue and target.

3 Hommel’s Experiment 1 to 3 focused on the local Simon effect, closely aligning with Stoffer’s attentional zooming hypothesis, whereas Experiment 4 to 6 addressed the global Simon effect under different cueing conditions.

4 Note: We also attempted to replicate Experiment 2 from Stoffer (1991), which used a spatial compatibility task requiring direct responses to the target’s spatial position. Due to procedural ambiguities in the Stoffer’s report of Experiment 2, those data are presented as supplementary material, allowing the current paper to maintain focus on attentional mechanisms as the source of the Simon effect.

5 Stoffer reported 200 trials per block (Stoffer, 1991, p. 131), however, this number is inconsistent with the reported six repetitions of 32 unique trial combinations, which would result in a total of 192 trials per block. Since the final analysis included 960 trials, 192 trials should have been included in one block.

6 Additionally, in line with the preregistration, we performed a separate analysis in which the response times outliers were identified as 1.5 times above the third quarter of the individual’s response distribution (Tukey, 1977). By using this criterium, a total of 3.2% of trials were filtered out as outliers. The analysis based on that data yielded identical results to analysis presented here.

7 In order to address a potential speed-accuracy trade-off in the data, data were additionally analysed using the inverse efficiency score (IES) (see Bruyer & Brysbaert (2011) combining response speed and accuracy into a single measure. The ANOVA with the five factors observed identical results, especially regarding the two Simon effects as shown by the interaction between Screen Position and Response, *F*_(1,26)_ = 47.34, *p* <.001, $\widehat n_G^2$ = 0.120, as well as Relative Position and Response, *F*_(1,26)_ = 11.54, *p* =.002, $\widehat n_G^2$ = 0.023, were significant, the dependency of the local Simon effect on Target Presentation shown by the interaction of Target Presentation, Relative Postion and Response, *F*_(1,26)_ = 51.73, *p* <.001, $\widehat n_G^2$ = 0.037, or Precue Type shown by the interaction of Precue Type, Relative Position and Response, *F*_(1,26)_ = 16.94, *p* <.001, $\widehat n_G^2$ = 0.012, as well as the five-way interaction, *F*_(1,26)_ = 8.89, *p* =.006, $\widehat n_G^2$ = 0.003.

8 Neither Stoffer (1991) nor Weeks et al. (1996) directly tested these small local Simon effects against 0, leaving the true reliability of the effect in this condition unclear.

9 While these correlations offer preliminary descriptive insight, their interpretation is limited by sample size, data aggregation, and known challenges in linking within-subject associations to underlying mechanisms (Haslbeck & Epskamp, 2024; Molenaar, 2004). They should thus be viewed as exploratory rather than conclusive.
