# Supplementary material for: Shifting and zooming through space … or not: The role of attention in spatial compatibility tasks - A replication of Stoffer (1991)
Source: Psychol Res. 2026 May 4;90(3):86. doi: 10.1007/s00426-026-02299-1 (PMC13139253; doi:10.1007/s00426-026-02299-1)
Supplement: Supplementary file 1 — (DOCX 18.9 KB) [file 426_2026_2299_MOESM1_ESM.docx]

**Supplementary material**

Experiment 2

The stimuli and setup for this experiment mirrored those of Experiment 2 in Stoffer's (1991) study. However, during the data analysis, we identified a discrepancy in the original report. Unlike Experiment 1, where participants classified the target as a square or rectangle, Stoffer (1991) only used the rectangle as a target stimulus in Experiment 2 and asked participants to respond based on the target’s spatial location (i.e. left or right part of the precue; see p. 133). Despite this, the paper reported that a five-factorial repeated measurement ANOVA was conducted, identical to the analysis used in Experiment 1. Those five factors were: Precue Type (large box vs. small boxes), Target Presentation (simultaneous vs. sequential), Screen Position (left or right), Relative Position (left or right), and Response (left or right), with the latter assumed to reflect the classification of the target as square or rectangle. However, since stimulus classification was omitted in Experiment 2, only four factors were left. Moreover, while Stoffer (1991) reports the spatial compatibility effect proper, which typically requires a compatible versus an incompatible mapping between (relative) target position and response. Instead, here, left stimuli within any precue (i.e., large or small box) always require a left response and vice versa, right stimuli require a right response independent of the screen position side, precluding a typical compatibility vs. incompatibility comparison as the mapping based on the relative position never changed (i.e, left stimuli requiring a right response). Thus, only one kind of spatial compatibility effect can be observed, i.e. the one based on the global screen position as the task-relevant feature.

Given the lack of clearance in the exact procedure of Experiment 2, i.e. whether indeed a fifth factor (for example, introducing a counter-balanced response mapping) was implemented but not reported) or whether the report of a fifth factor in the analysis was just a reporting error, we felt that reporting Experiment 2 would not make a significant contribution to the ongoing debate regarding the attentional mechanisms underlying the Simon effect that we wanted to contribute to by our research. Therefore, for the sake of completeness, we report our results of Experiment 2 here.

**Method**

**Subjects**. 30 students took part in the study, of which 24 were female (M = 24.53, SD = 4.25). All participants gave consent prior to the experiment, and ethical approval was obtained from the local ethics committee at the University of Hildesheim. As compensation, participants received course credit for their participation.

**Material**. Stimuli were identical to those of Experiment 1, apart from the fact that the rectangle was the only target used as a stimulus.

**Design**. A four‐way within-subjects factorial design was employed to examine the spatial compatibility effect proper in Experiment 2. The following factors were manipulated: Precue Type (small boxes vs. large box), Target Presentation (simultaneous vs. sequential), Screen Position (left vs. right), and Response (left vs. right). This manipulation resulted in 16 experimental conditions, for each of which, 60 trials were conducted, resulting in 960 experimental trials in total. The key dependent variables were the reaction times and the accuracy of responses.

**Procedure**. The procedure followed the same steps as in Experiment 1, with the exception of the instructions. Participants were informed that their task was to respond based on the location of the imperative stimulus. Specifically, they were instructed to respond as quickly as possible to whether the stimulus was displayed on the precue’s relative left or right position (i.e, in the left or right small box or on the left or right position within the single large box), ignoring the precue’s screen position side.

**Results**

**Data analysis**

**Response time analyses**. A total of 3.09% of trials were excluded from further analysis due to incorrect responses; no reaction time outliers were identified. A repeated-measures ANOVA was conducted with the following within-subject factors^^[[1]](#footnote-1)^^: Precue Type (large box vs. small boxes), Target Presentation (simultaneous vs. sequential), Screen Position (left, right), and Response (left, right), which is equivalent to the Relative Position of the target within the precue. Results are shown in Supplementary Table 1.

The corresponding ANOVA revealed three main effects. First, there was a marginally main effect of Precue Type, *F*_(1,29)_ = 4.09, *p* = .052, $\eta_{p}^{2}$ = .124, reporting faster responses for small boxes (389 ms ± 8 SEM) compared to the large box (392 ms ± 9 SEM); Second, a significant main effect of Response/Relative Position emerged, *F*_(1,29)_ = 6.21, *p* = .019, $\eta_{p}^{2}$ = .176, indicating faster reactions for right responses (385 ms ± 8 SEM) [equivalent with stimuli’s right relative position in regard to any precue type] compared to left responses (396 ms ± 10 SEM). Third, a significant main effect of Target Presentation was found, *F*(1,29) = 608.44, *p* < .001, $\eta_{p}^{2}$ = .955, showing faster reactions for sequential target presentation (335 ms ± 8 SEM) compared to simultaneous ones (446 ms ± 10 SEM).

Additionally, there was a significant interaction between Precue Type and Target Presentation, *F*_(1,29)_ = 608.44, *p* < .001, $\eta_{p}^{2}$ = .955. Post-hoc tests revealed significant differences between all pair-wise comparisons, *t*_(29)_ ≥ 3.87, *p* ≤ .001, except between the small and the large boxes under simultaneous target presentation, where no significant difference was observed. Of particular interest to this research, the interaction between Screen Position and Response was also significant, *F*_(1,29)_ = 10.99, *p* = .002, $\eta_{p}^{2}$ = .275, reflecting a spatial compatible effect proper (9 ms ± 3 SEM) with faster responses on compatible trials (386 ms ± 8 SEM) compared to incompatible trials (395 ms ± 9 SEM).

Moreover, the spatial compatibility effect proper interacted further with the Precue Type as indicated by the interaction of Precue Type, Screen Position and Response, *F*_(1,29)_ = 13.33, *p* = .001, $\eta_{p}^{2}$ = .315, showing differences in the size of the spatial compatibility effect between the large box (15 ms ± 4 SEM) and the small boxes (3 ms ± 3 SEM) as precues, *t*_(29)_ = 3.65, *p* < .001.

The spatial compatibility effect proper was also modulated by the Target Presentation as shown by the interaction of Target Presentation, Screen Position, and Response, *F*_(1,29)_ = 86.06, *p* < .001, $\eta_{p}^{2}$ = .748. The interaction revealed differences in the size of the spatial compatibility effect proper between the simultaneous (31 ms ± 5 SEM) and sequential target presentation (-13 ms ± 2 SEM), *t*_(29)_ = 9.28, *p* < .001.

The 4-way interaction between Precue Type, Target Presentation, Screen Position, and Response was also significant, *F*_(1,29)_ = 25.81, *p* < .001, $\eta_{p}^{2}$ = .471. The spatial compatibility effect propers were significantly different from 0 under all combinations of Precue Type and Target Presentation, t(29) ≥ 3.60, p ≤ .001 (see also Supplementary Table 2).

Supplementary Table 1

Mean reaction times (in ms) as a function of Precue Type, Target Presentation, Screen Position and Response [corresponding to Relative Position] in Experiment 2.

| Precue Type | Target Presentation | Screen  Position | Response | Mean reaction time (± SEM) |
| --- | --- | --- | --- | --- |
| One Large box | Simultaneous | left | left | 433 ms (± 11) |
|  |  |  | right | 462 ms (± 11) |
|  |  | right | left | 473 ms (± 14) |
|  |  |  | right | 417 ms (± 9) |
|  | Sequential | left | left | 350 ms (± 9) |
|  |  |  | right | 329 ms (± 9) |
|  |  | right | left | 336 ms (± 9) |
|  |  |  | right | 339 ms (± 7) |
| Two small boxes | Simultaneous | left | left | 444 ms (± 10) |
|  |  |  | right | 455 ms (± 10) |
|  |  | right | left | 458 ms (± 11) |
|  |  |  | right | 428 ms (± 9) |
|  | Sequential | left | left | 344 ms (± 9) |
|  |  |  | right | 318 ms (± 7) |
|  |  | right | left | 331 ms (± 9) |
|  |  |  | right | 332 ms (± 7) |

Supplementary Table 2

Global Spatial compatibility effect propers (in ms; SEM in parentheses) in Experiment 2.

| Precue Type | Target Presentation | Screen Position Compatibility effect |
| --- | --- | --- |
| Large box | Simultaneous | 43 ms (± 6) |
|  | Sequential | -12 ms (± 3) |
| Small boxes | Simultaneous | 20 ms (± 4) |
|  | Sequential | -13 ms (± 3) |

1. According to Stoffer (1991) a 5-factorial ANOVA was calculated. However, Experiment 2 did not require a stimulus classification as in Experiment 1. Therefore, there are only four factors left in the design. The analysis carried out by Stoffer (1991) is rather unclear as it was reported that the interaction between Cue Type, Target Presentation, Relative Position [relative position of stimulus within precue] and Response [‘position of response key’] was not significant. Given that only one kind of stimulus was used requiring a classification of its relative position as left or right within the precue, the factor ‘Relative Position’ corresponds to ‘Response’. Thus, it is rather likely that one factor was incorrectly named. Accordingly, results based on the Relative Position probably report effects based on the Screen Position. [↑](#footnote-ref-1)
